# Dietary and Serum Antioxidants Associated with Prostate-Specific Antigen for Middle-Aged and Older Men

**DOI:** 10.3390/nu15153298

**Published:** 2023-07-25

**Authors:** Hui-Yi Lin, Xiaodan Zhu, Alise J. Aucoin, Qiufan Fu, Jong Y. Park, Tung-Sung Tseng

**Affiliations:** 1 Biostatistics Program, School of Public Health, Louisiana State University Health Sciences Center, New Orleans, LA 70112, USA; 2 School of Medicine, Louisiana State University Health Sciences Center, New Orleans, LA 70112, USA; aauco4@lsuhsc.edu; 3 Department of Cancer Epidemiology, Moffitt Cancer Center & Research Institute, Tampa, FL 33612, USA; jong.Park@moffitt.org; 4 Behavior and Community Health Sciences Program, School of Public Health, Louisiana State University Health Sciences Center, New Orleans, LA 70112, USA; ttseng@lsuhsc.edu

**Keywords:** PSA, prostate cancer, oxidative stress, antioxidant, albumin, vitamin D

## Abstract

High prostate-specific antigen (PSA) levels can indicate potential prostate problems and are a warning sign of prostate cancer. The impact of antioxidants on the PSA of generally healthy men is understudied. This study aims to evaluate 14 dietary and endogenous antioxidants associated with PSA levels for United States (US) men. We assessed 7398 men using the 2003–2010 US population-based National Health and Nutrition Examination Survey (NHANES). The PSA levels were categorized into three groups: Normal, borderline, and elevated levels. We performed analyses for middle-aged and older groups aged 40–64.9 and ≥65, respectively. The weighted multinomial regressions were performed to evaluate antioxidants associated with the PSA status. For results, 0.3% and 3.4% of middle-aged and older men, respectively, had elevated PSA (>10 ng/mL). Men with a higher serum albumin level had a lower risk of an elevated PSA, adjusting for age. The magnitude of albumin’s impact on PSA is larger in middle-aged men than in older men (OR of elevated PSA = 0.82 and 0.90, respectively, interaction *p* = 0.002). Other antioxidants are not associated with PSA. Our findings support men with low serum albumin tend to have an elevated PSA level, so related interventions can be considered to decrease PSA for maintaining prostate health.

## 1. Introduction

The Prostate-Specific Antigen (PSA), a serine protease secreted by prostatic epithelial cells, is the most commonly used biomarker in detecting and monitoring prostate health [1]. An elevated PSA level in the blood can indicate prostate-related diseases, such as prostatitis, benign prostatic hyperplasia (BPH), and prostate cancer. Prostate cancer cells often produce higher levels of PSA, resulting in elevated levels in the blood. It is well-known that the probability of prostate cancer increases with the level of PSA. It has been shown that men whose PSA falls within the borderline range (4–10 ng/mL) have an approximate 25% chance of having prostate cancer, and men with a PSA level exceeding 10 ng/mL have a probability greater than 50% [2]. In addition, high PSA levels could be affected by other factors, such as aging, infection, genetics, dietary factors, and antioxidants [3,4].

It is commonly known that aging plays an essential role in increasing serum PSA levels [5,6,7,8]. For instance, a study compared PSA levels in men aged 60 and above and found a significant increase in PSA levels with increasing age [5,6]. The serum PSA level increases by approximately 0.04 ng/mL per year in healthy men over 60 [5]. Furthermore, prostate cancer is a heterogeneous disease. Early-onset prostate cancer, that is, prostate cancer diagnosed at an early age, has been considered a distinct phenotype compared with regular prostate cancer diagnosed at an older age. This is because men with early-onset prostate cancer tend to have more aggressive prostate cancer, leading to a worse prognosis [9,10]. For example, men aged 35–44 with high-grade prostate tumors had a 1.7 times higher risk of prostate cancer-related deaths than men aged 65–74 [11]. In addition, there is an increasing trend of prostate cancer incidence in young and middle-aged men, although most men are diagnosed with prostate cancer at an older age [10]. Therefore, evaluating risk factors associated with an elevated PSA for younger men is gaining more interest.

In addition to aging, antioxidants are modifiable factors that may affect PSA levels. Antioxidant defense mechanisms are engaged to maintain the oxidation-reduction (redox) status, the balance between the levels of oxidants and antioxidants [12,13]. The antioxidants include endogenous and dietary antioxidants, which work together to retain redox homeostasis [14,15]. Endogenous antioxidants, such as albumin, bilirubin, and uric acid, are products of the body’s metabolism and can protect against oxidative damage. On the other hand, dietary antioxidants, such as vitamins C and D, are substances in foods that can also help prevent oxidative damage.

The preventive role of antioxidants in developing BPH and prostate cancer, closely related to PSA levels, remains inconclusive. Some studies have supported the preventive role of antioxidants in developing BPH and prostate cancer [16,17]. For example, the Mediterranean diet, characterized by a high intake of fruits, vegetables, fish, and olive oil, is rich in antioxidants and has been shown to have a protective effect on prostate cancer [4,18]. Furthermore, some studies showed that nutrients such as vitamins A, C, D, and E and selenium are known to act as antioxidants, which may help reduce the risk of prostate cancer development [16,19,20]. Moreover, it has also been shown that men with prostate cancer typically display greater oxidative stress levels than men with BPH and asymptomatic inflammatory prostatitis [21]. However, other studies have not reported the protective role of antioxidants (such as vitamins C, D, E, and selenium) in elevated PSA levels and the development of prostate cancer [22,23,24]. Most antioxidant-related studies are for BPH or prostate cancer patients, so the associations between antioxidants and PSA remain unclear for generally healthy and younger men. Therefore, it is important to evaluate the modifiable antioxidants associated with PSA for different age groups. To address these issues, the objective of this study is to assess a wide range of 14 antioxidants, including 3 serum endogenous antioxidants and 11 dietary antioxidants, associated with PSA in middle-aged and older men using large-scale population-based data.

## 2. Materials and Methods

### 2.1. Study Population

This study included 7397 men from the population-based National Health and Nutrition Examination Survey (NHANES), an open-access, nationally representative survey from 2003 to 2010. The inclusion criteria are male participants aged 40 years older enrolled in PSA laboratory testing and with valid data for at least 1 of the 14 selected antioxidants. In addition, individuals that reported having any of the following conditions were not eligible for PSA testing in the NHANES: Current infection or inflammation of the prostate gland, a rectal exam in the past week, prostate biopsy in the past month, cystoscopy in the past month, or a history of prostate cancer.

### 2.2. Data Collection and Measurements

This study used the total PSA concentration levels to measure PSA levels. PSA immunoassays were performed on blood specimens using the Hybritech tests (Beckman Coulter, Fullerton, CA, USA). A total of 14 antioxidants (endogenous and dietary antioxidants) were tested. Among them, 3 endogenous antioxidants (bilirubin, albumin, and uric acid) were measured in the serum samples. For these serum biomarkers, the detailed procedures and analytic methods are described in the NHANES Description of Laboratory Methodology section. In addition, we tested 11 dietary antioxidants: Vitamins A, B2, C, D, and E, alpha-carotene, selenium, lycopene, lutein + zeaxanthin, beta-cryptoxanthin, and folate. For dietary antioxidants, two 24-h dietary recall interviews were applied, and the averages of the two 24-h dietary measures were used. Of these 14 antioxidants, 13 were available in the 2003–2010 NHANES data; vitamin D was only available from 2007–2010, resulting in a smaller sample size compared with the others.

### 2.3. Statistical Analyses

The outcome of this study is the 3-group PSA status: Normal (≤4 ng/ml), borderline (4.01–10 ng/mL), and elevated levels (>10 ng/mL) [2]. The antioxidants associated with PSA may be varied by age, so all analyses were performed separately for the 2 age groups: The middle-aged group (40–64.9 years old) and the older group (≥65 years old). In addition, demographic factors (age, race, and income), BMI status, smoking status, and the number of days using alcohol during the past 12 months listed in Table 1 were evaluated. For income, we used the income-to-poverty ratio, which is the ratio of the family’s total income compared with the US federal poverty threshold, which considers the family size. The income-to-poverty ratio is in the range of 0 (no income) to 5 (≥5 times the US federal poverty level). For evaluating these demographic and behavioral factors associated with PSA levels, the Rao-Scott chi-square tests were applied for categorical factors, and weighted linear regressions were applied for continuous factors. In addition, we tested one antioxidant at a time using the weighted multinomial logistic models with the 3-group PSA status as the outcome. Both unadjusted and adjusted logistic models were applied to evaluate associations between the antioxidants and PSA status. Due to the small sample size for the elevated PSA groups, we limited the number of adjusting covariates for model stability. Thus, only significant factors with a *p*-value < 0.05 in the multivariable model with the adjusting factors were included in the multivariable models. Therefore, age was adjusted in the multivariable models for the middle-aged group, and age and race were adjusted for the older group. To test whether age modifies the association between albumin and PSA, an interaction of the binary age factor and serum albumin was tested in a multinomial logistic model based on a combined set with the two age groups adjusted for race. The statistically significant level is 0.05. All analyses were weighted to consider the complex NHANES sampling design using SAS 9.4 (SAS Institute, Inc., Cary, NC, USA). The weighting analytical procedures followed the NHANES suggested instruction.

## 3. Results

The demographic and behavioral factors by the PSA levels (normal, borderline, and elevated levels) and age (middle-aged and older groups) are listed in Table 1, Tables S1 and S2. For the middle-aged group, 2.8% had a borderline PSA (4.01–10 ng/mL) and 0.3% had an elevated PSA (>10 ng/mL). For the older group, there are 14.1% with a borderline PSA and 3.4% with an elevated PSA. For demographic factors, age and race were significantly associated with PSA levels for both middle-aged and older groups. For middle-aged men, age (*p* < 0.001 based on a weighted linear regression), race (*p* = 0.023 based on the Rao-Scott chi-square tests), and income (*p* < 0.001 based on the Rao-Scott chi-square tests) were significantly associated with PSA based on the bivariate analyses, but the associations between PSA and other factors (BMI status, smoking, and alcohol) were not significant. As shown in Table 1 and Table S1, age acceleration is significant with a borderline PSA but not an elevated PSA for middle-aged men based on the weighted multinomial logistic model adjusting for age (OR of borderline PSA = 1.19, *p* < 0.001). For race, non-Hispanic Black Middle-aged men had a higher risk of elevated PSA than non-Hispanic white men (OR = 7.09, *p* = 0.009) in the unadjusted model. However, race became insignificant when considering age and income. For income, men with higher income had a lower risk of elevated PSA levels based on the unadjusted and adjusted results. Due to the small sample size for the elevated PSA groups for the middle-aged group, we included age, the most significant predictor (Table S1), in the multivariable weighted multinomial logistic model for the adjusting purpose. For older men, only age (*p* = 0.001) and race (*p* = 0.002) were significantly associated with PSA, while the association between BMI status to PSA was marginally significant (*p* = 0.053). Age was positively associated with PSA (OR = 1.05 and 1.07, and *p* = 0.005 and 0.021 for borderline and elevated PSA levels, respectively). In addition, non-Hispanic Blacks had a higher risk of borderline (OR = 1.82, *p* = 0.002) and elevated PSA levels (OR = 3.17, *p* < 0.001) than non-Hispanic Whites aged 65 and above.

For middle-aged men, the results of antioxidants associated with PSA analyzed using the weighted multinomial logistic models are shown in Table 2 and Table 3. Among the 14 antioxidants, serum albumin (*p* < 0.001) and vitamin D (*p* < 0.001) were significantly associated with PSA for middle-aged men based on the univariate results. Men with a higher PSA tended to have a lower serum albumin level. The means of serum albumin levels were 43.4, 42.5, and 40.6 g/L for men with a normal, borderline, and elevated PSA, respectively. Middle-aged men with borderline or elevated PSA had significantly lower albumin levels compared with men with a normal PSA (odds ratio (OR) = 0.91 and 0.82 for albumin per 1 g/L, both *p* < 0.001) based on the unadjusted results. After adjusting for age, serum albumin associated with a borderline PSA became insignificant, but the inverse association between serum albumin and an elevated PSA remains significant (OR = 0.82 for albumin per 1 g/L, *p* < 0.001). In addition, vitamin D was marginally associated with PSA in the unadjusted and adjusted models. Based on the unadjusted results, middle-aged men with an elevated PSA had significantly lower vitamin D levels than men with a normal PSA (3.0 vs. 5.5 mcg, OR = 0.80, *p* = 0.063). Similarly, vitamin D was associated with an elevated PSA but not a borderline PSA after adjusting for age (OR = 0.80, *p* = 0.068).

For older men, the results of antioxidants associated with PSA analyzed using the weighted multinomial logistic models are shown in Table 4 and Table 5. For older men, albumin (*p* < 0.001) and vitamin A (*p* = 0.011) were significantly associated with PSA (Table 4). In addition, the inverse associations of three dietary antioxidants (alpha-carotene, Beta-cryptoxanthin, and folate) and PSA were marginally significant (*p* = 0.072, 0.057, and 0.055, respectively). After adjusting for age and race (Table 5), only albumin was significantly associated with PSA, but the associations between other antioxidants and PSA were insignificant. As shown in Table 4 and Table 5, older men with an elevated PSA had significantly lower albumin compared with men with a normal PSA in both unadjusted and adjusted models (42.0 vs. 40.6 g/L, adjusted OR of an elevated PSA = 0.90, *p* < 0.001).

**Figure 1:**
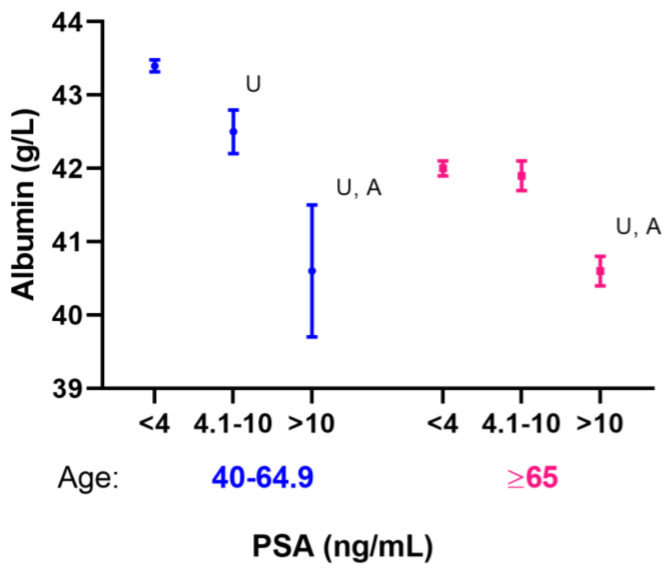
Serum albumin distribution by age and PSA status. Note: Means and 95% confidence intervals are shown. U: Unadjusted *p*-value < 0.001; A: Adjusted *p*-value < 0.001 compared with the normal PSA group (<4 ng/mL), adjusted age for the middle-aged group, and age and race for the older group.

The effect size in terms of OR values of an elevated PSA differs between the middle-aged and older groups, so we were also interested in testing whether this difference was statistically significant. Therefore, an interaction test of age and albumin was performed in the combined dataset with both age groups. As shown in Figure 1, the impact magnitude of albumin on PSA is significantly larger for middle-aged men than for older men (interaction *p* = 0.002). Thus, this supports that age is a significant modifier in modifying the association between albumin and PSA. The adjusted OR of an elevated PSA was 0.82 and 0.90 for 1 g/L of albumin for middle-aged and older men, respectively (Table 3 and Table 5).

## 4. Discussion

This study showed that serum albumin levels were inversely associated with PSA for both middle-aged (40–64.9 years) and older men (≥65 years) in the unadjusted and adjusted models. However, these associations varied by the PSA levels and age. Higher serum albumin levels had a lower risk of an elevated PSA (>10 ng/ml) but not with borderline PSA (range 4.1–10 ng/mL). Moreover, age significantly modified the association between albumin and elevated PSA levels (interaction *p* = 0.002), with a larger effect in middle-aged men than in older men. Furthermore, vitamin D was marginally associated with lower elevated PSA levels in middle-aged men in both unadjusted and adjusted models, suggesting a potential benefit of vitamin D in reducing PSA for this age group. For other antioxidants, none showed significant associations with PSA in the adjusted results, although some dietary antioxidants showed promising results in the unadjusted models. For the older men, vitamin A and folate showed a significant protective effect on an elevated PSA in the unadjusted models, but these associations became insignificant in the adjusted models.

For serum albumin levels, most participants in this study (98.5% for the middle-aged and 98.4% for older men) had serum albumin levels in the normal range, 35–50 g/L [25]. It should be noted that the mean serum albumin levels for men with an elevated PSA (mean = 40.6 for both middle-aged and older men) were lower than that for men with a normal PSA, although still within the normal range. Serum albumin has numerous functions for contributing to health. Among them, albumin’s antioxidant properties play a crucial role in critical pathologies such as cancer by balancing the plasma redox state [25]. Albumin is the most abundant plasma protein in the human body. Therefore, serum albumin is commonly used as a biomarker for testing nutritional status [26]. Low levels of serum albumin are commonly observed in malnourished individuals. Serum albumin levels can be elevated by consuming a well-balanced diet rich in protein, such as lean meats, fish, eggs, nuts, and dairy products. In addition to nutritional status, serum albumin can be impacted by multiple factors, such as inflammation, immune function, infection, liver and kidney function, and chronic diseases [26,27]. A low serum albumin level induces less albumin-binding testosterone, which may lead to an increased risk of prostate cancer incidence, recurrence, progression, and poor outcomes [27,28,29].

Serum albumin associated with prostate health is supported by other studies. One study reported a non-linear relationship between serum albumin and PSA. When serum albumin exceeds 41 g/L, there is an inverse association between albumin and PSA for men aged 40 and older [30]. Moreover, another study showed that men with a high albumin level tended to have a low PSA and a lower risk of advanced prostate cancer [31]. A clinical trial followed 4770 men over 7 years and reported that a diet high in protein may reduce the risk of symptomatic BPH [32]. Additionally, several indices involved with serum albumin have been shown to predict prostate cancer risk and prognosis. The HALP score comprises four biomarkers (hemoglobin, albumin, lymphocyte, and platelet) and has been used as a prognostic marker for many cancers, including prostate cancer. For example, a high HALP score is associated with better prostate cancer prognosis in terms of progression-free survival [33]. Moreover, it has been shown that the albumin levels were significantly lower in prostate cancer patients than those with BPH (*p* = 0.0001) [34]. Another albumin-related index is the prognostic nutritional index (PNI), which has been used as a prognostic marker for predicting survival in prostate cancer patients. PNI is calculated based on the serum albumin level and peripheral blood lymphocyte count and is commonly used as an indicator of systemic inflammation and immune and nutritional status [35,36]. Prostate cancer patients with high pre-treatment PNI values, corresponding to high serum albumin, have better response rates with treatments and survival outcomes, including prostate cancer progression-free survival and overall survival [37]. Another study also indicated that prostate cancer patients with low serum albumin levels and high body mass index (BMI) had a high risk of death [38]. These findings support the potential role of albumin and endogenous antioxidants on PSA and related prostate health.

Vitamin D also plays a key role in many diseases, including prostate cancer. Vitamin D, which is involved in cell proliferation, differentiation, and apoptosis, may protect against various diseases, including prostate cancer [39]. Furthermore, serum vitamin D metabolites are bound to vitamin D binding protein (DBP) and albumin [40]. Therefore, low albumin levels lead to a reduction in serum vitamin D levels [41]. Indeed, prostate cancer patients have significantly lower serum 25(OH)-vitamin D compared with men without prostate cancer (16.2 vs. 23.2 ng/mL, respectively, *p* < 0.001) [42]. In addition, low-grade prostate cancer patients managed with active surveillance who took vitamin D supplements are two times more likely to have a negative PSA trend [43]. However, some studies did not support the associations between vitamin D and PSA [44]. Therefore, vitamin D may impact reducing the risk of prostate cancer, but the results remain inconclusive.

A strength of this study is that it utilizes US national survey data with appropriate sampling weighting, which allows for the generalization of the results to the US population. It also has a well-designed statistical plan, with three-group PSA levels based on clinically meaningful cut-points and sub-group analyses. In addition, it is novel that this study reports the age modification effect on the association between serum albumin and PSA. This provides valuable information that can inform age-specific health interventions and clinical practices. Despite these strengths, there are some limitations to this study. First, the sample sizes for the elevated PSA groups are small, which limits the statistical power of this study and restricts the number of adjusting factors in modeling. Second, no causality can be presumed between antioxidants and PSA due to the nature of a cross-sectional study. Third, there may be a recall bias for the self-reported dietary interview, and the two-day dietary may not represent the general dietary pattern for interviewers.

## 5. Conclusions

Our findings support that men with a higher serum albumin level had a lower risk of an elevated PSA, and this effect is more prominent in middle-aged men than in older men. A potential benefit of vitamin D in reducing PSA was also observed for middle-aged men but not for older men. However, bilirubin, uric acid, and the other 10 selected dietary antioxidants were not associated with PSA. Therefore, healthcare providers may suggest a well-balanced diet rich in protein for men to increase their serum albumin levels and lower PSA levels. In order to elucidate the causal relationship between albumin and PSA for generally healthy men, longitudinal studies will be needed.

## Figures and Tables

**Table 1:** Participants’ demographic and behavioral factors by age and PSA status.

		Middle-Aged Men (Age: 40–64.9, n = 3651)				Older Men (Age: ≥65, n = 2119)			
	Total	PSA ≤ 4	PSA: 4.01–10	PSA > 10	*p*-Value ^b^	PSA ≤ 4	PSA: 4.01–10	PSA > 10	*p*-Value ^b^
	N (wt %) ^a^	N (wt %) ^a^	N (wt %) ^a^	N (wt %) ^a^		N (wt %) ^a^	N (wt %) ^a^	N (wt %) ^a^	
Total	5770	3515 (97.0)	120 (2.8)	16 (0.3)		1716 (82.5)	316 (14.1)	87 (3.4)	
Age	56.2 ± 0.2	50.7 ± 0.2	57.6 ± 0.7	53.0 ± 1.9	**<0.001**	72.5 ± 0.2	74.0 ± 0.5	74.8 ± 1.0	**0.001**
Race									
Non-Hispanic white	3138 (77.3)	1701 (97.1)	55 (2.8)	3 (0.1)	**0.023**	1128 (83.1)	205 (13.9)	46 (3.0)	**0.002**
Non-Hispanic Black	1057 (9.2)	722 (96.6)	26 (2.5)	7 (0.9)		222 (72.0)	58 (20.5)	22 (7.5)	
Other	1575 (13.5)	1092 (96.6)	39 (2.9)	6 (0.5)		366 (84.5)	53 (11.3)	19 (4.3)	
Income to poverty ratio ^c^									
≤1	864 (9.5)	556 (96.9)	14 (1.8)	6 (1.3)	**<0.001**	225 (78.2)	53 (19.3)	10 (2.4)	0.494
1.1–4	2876 (46.2)	1586 (97.9)	46 (2.0)	5 (0.1)		1003 (81.9)	190 (14.7)	46 (3.4)	
>4	1633 (44.3)	1140 (96.4)	50 (3.4)	4 (0.2)		361 (83.9)	58 (12.5)	20 (3.5)	
Body Mass Index (BMI)									
<25	1320 (21.3)	750 (97.1)	24 (2.8)	2 (0.1)	0.218	415 (77.7)	95 (17.7)	34 (4.5)	0.053
25–29.9	2397 (43.1)	1442 (96.3)	54 (3.4)	9 (0.3)		728 (83.0)	133 (14.2)	31 (2.8)	
≥30	1963 (35.6)	1287 (97.8)	41 (2.0)	5 (0.2)		533 (86.1)	79 (10.8)	18 (3.1)	
Smoking ^d^									
Never	2196 (40.0)	1458 (96.6)	54 (3)	10 (0.4)	0.190	524 (79.3)	114 (16.2)	36 (4.5)	0.236
Former	2274 (37.7)	1040 (96.7)	42 (3.2)	3 (0.1)		988 (84.3)	162 (12.8)	39 (2.9)	
Current	1297 (22.3)	1016 (97.8)	24 (1.8)	3 (0.3)		203 (82.7)	39 (14.6)	12 (2.8)	
Days of alcohol usage in the past 12 months									
≤3	815 (17.1)	449 (94.3)	21 (5.0)	4 (0.7)		280 (84.6)	49 (12.7)	12 (2.7)	
4–50	1094 (27.3)	767 (97.6)	18 (2.0)	6 (0.3)		248 (85.5)	44 (11.7)	11 (2.8)	
>50	2130 (55.6)	1424 (97.1)	51 (2.7)	4 (0.2)	0.069	531 (81.1)	96 (15.9)	24 (2.9)	0.578

^a^ weighted % is based on the NHANES sampling weights. ^b^ *p*-values are based on the univariate weighted linear regression for age and the Rao-Scott chi-square test for categorical factors. ^c^ family’s total income compared with the US federal poverty threshold, which considers the family size. ^d^ Never smoking is defined as smoked <100 cigarettes in life, and former smoking is defined as smoked ≥100 cigarettes in life but did not smoke currently.

**Table 2:** Levels of antioxidants by PSA status for middle-aged men aged 40–64.9.

Factors	Total Mean ± SE ^a^ (n = 3651)	PSA ≤ 4 Mean ± SE ^a^ (n = 3515)	PSA: 4.01–10 Mean ± SE ^a^ (n = 120)	PSA: >10 Mean ± SE ^a^ (n = 16)	*p*-Value ^b^
**Endogenous antioxidants**					
Bilirubin (umol/L)	14.28 ± 0.12	14.27 ± 0.1	14.7 ± 0.7	12.5 ± 0.8	0.077
Albumin (g/L)	43.39 ± 0.08	43.4 ± 0.08	42.5 ± 0.3	40.6 ± 0.9	<0.001
Uric acid(umol/L)	363.78 ± 1.94	364.00 ± 2.0	357.9 ± 10.5	343.9 ± 21.5	0.514
**Dietary antioxidants**					
Vitamin A, RAE ^c^ (mcg)	687.9 ± 12.2	686.9 ± 12.3	720.1 ± 52.8	707.9 ± 170	0.815
Vitamin B2 (mg)	2.6 ± 0.03	2.6 ± 0.03	2.5 ± 0.2	2.5 ± 0.4	0.589
Vitamin C (mg)	92.4 ± 2.1	92.0 ± 2.1	103.5 ± 9.5	112.3 ± 43.6	0.443
Vitamin D (mcg)	5.6 ± 0.2 (n = 2131)	5.5 ± 0.2 (n = 2051)	7.2 ± 1.7 (n = 71)	3 ± 0.7 (n = 9)	<0.001
Vitamin E (mg)	8.6 ± 0.1	8.6 ± 0.1	8.4 ± 0.4	9.6 ± 2	0.759
Alpha carotene (mcg)	405.3 ± 18.1	403.6 ± 18.4	468.7 ± 97.7	367.7 ± 168.9	0.789
Selenium (mcg)	132.1 ± 1.4	132.1 ± 1.4	131.3 ± 5.7	126.3 ± 13	0.869
Lycopene (mcg)	6767.1 ± 227.2	6761.9 ± 242.8	6869.7 ± 1052.5	7631 ± 2463.2	0.939
Lutein + zeaxanthin (mcg)	1581.4 ± 66	1582.1 ± 68.1	1501.8 ± 149.8	2174.9 ± 854.5	0.656
Beta-cryptoxanthin (mcg)	115.9 ± 5.0	115.4 ± 5.1	132.2 ± 20.6	113.7 ± 44.4	0.750
Folate, DFE ^c^ (mcg)	467.8 ± 6.6	467.4 ± 6.7	473.7 ± 22	544.7 ± 78.2	0.570

^a^ Weighted mean± standard error based on the NHANES sampling weights. ^b^ Compared antioxidant levels among the three PSA groups using the univariate weighted multinomial logistic model. ^c^ RAE: Retinol activity equivalents, DFE: Dietary folate equivalents.

**Table 3:** Antioxidants associated with PSA status for middle-aged men aged 40–64.9.

	Unadjusted ^a^		Adjusted ^a^	
Factors (Unit)	PSA 4–10 vs. PSA < 4 OR (95% CI)	PSA ≥ 10 vs. PSA < 4 OR (95% CI)	PSA 4–10 vs. PSA < 4 OR (95% CI)	PSA ≥ 10 vs. PSA < 4 OR (95% CI)
**Endogenous antioxidants**				
Bilirubin (umol/L)	1.02 (0.97, 1.07)	0.91 (0.82, 1.01)	1.03 (0.98, 1.08)	0.91 (0.82, 1.01)
Albumin (g/L)	0.91 (0.87, 0.96) ***	0.82 (0.75, 0.88) ***	0.96 (0.91, 1.01)	0.82 (0.76, 0.89) ***
Uric acid, per 10 umol/L	0.99 (0.95, 1.03)	0.96 (0.89, 1.05)	0.99 (0.95, 1.03)	0.96 (0.89, 1.05)
**Dietary antioxidants**				
Vitamin A, RAE ^b^, per 100 mcg	1.01 (0.98, 1.04)	1.01 (0.92, 1.1)	1.01 (0.98, 1.04)	1.01 (0.92, 1.1)
Vitamin B2 (mg)	0.90 (0.69, 1.17)	0.91 (0.52, 1.59)	0.97 (0.75, 1.27)	0.93 (0.52, 1.64)
Vitamin C (mg), per 10 mg	1.01 (0.99, 1.03)	1.02 (0.96, 1.09)	1.02 (0.99, 1.04)	1.02 (0.96, 1.09)
Vitamin D (mcg)	1.04 (0.98, 1.1)	0.8 (0.63, 1.01) ^#^	1.04 (0.99, 1.10)	0.80 (0.63, 1.02)^#^
Vitamin E (mg)	0.99 (0.96, 1.02)	1.03 (0.93, 1.14)	1.01 (0.98, 1.04)	1.03 (0.94, 1.14)
Alpha-carotene, per 100 mcg	1.01 (0.99, 1.03)	0.99 (0.93, 1.06)	1.01 (0.98, 1.03)	0.99 (0.93, 1.06)
Selenium (mcg), per 10 mcg	1.00 (0.96, 1.04)	0.98 (0.89, 1.07)	1.03 (0.99, 1.07)	0.99 (0.89, 1.1)
Lycopene, per 1000 mcg	1.00 (0.97, 1.03)	1.01 (0.96, 1.06)	1.01 (0.98, 1.04)	1.01 (0.96, 1.06)
Lutein + zeaxanthin, per 1000 mcg	0.99 (0.94, 1.04)	1.06 (0.95, 1.18)	0.98 (0.92, 1.04)	1.06 (0.94, 1.18)
Beta-cryptoxanthin, per 100 mcg	1.03 (0.97, 1.1)	1.00 (0.79, 1.25)	1.02 (0.96, 1.08)	0.99 (0.81, 1.23)
Folate, DFE ^b^, per 100 mcg	1.01 (0.93, 1.11)	1.13 (0.94, 1.35)	1.05 (0.97, 1.15)	1.14 (0.96, 1.35)

^#^ 0.05 < *p*-value < 0.1; ***: *p* < 0.001 based on multinomial logistic models. ^a^ weighted multinomial logistic model with and without adjusting for age; OR: Odds ratio; CI: Confidence interval. ^b^ RAE: Retinol activity equivalents, DFE: Dietary folate equivalents.

**Table 4:** Levels of antioxidants by PSA status for older men aged ≥65.

Factors (Unit)	Total Mean ± SE ^a^ (n = 2119)	PSA: 1–4 Mean ± SE ^a^ (n = 1716)	PSA: 4.01–10 Mean ± SE ^a^ (n = 316)	PSA: >10 Mean ± SE ^a^ (n = 87)	*p*-Value ^b^
**Endogenous antioxidants**					
Bilirubin (umol/L)	14.9 ± 0.2	15.0 ± 0.2	14.9 ± 0.4	14.3 ± 0.4	0.298
Albumin (g/L)	41.9 ± 0.1	42.0 ± 0.1	41.9 ± 0.2	40.6 ± 0.2	<0.001
Uric acid(umol/L)	368.2 ± 2.3	366.7 ± 2.4	373.7 ± 6.2	381.5 ± 20.3	0.392
**Dietary antioxidants**					
Vitamin A, RAE ^c^ (mcg)	753.7 ± 20.4	757.7 ± 22.6	766.2 ± 57.4	606 ± 48.2	0.011
Vitamin B2 (mg)	2.4 ± 0.04	2.4 ± 0.04	2.3 ± 0.1	2.2 ± 0.2	0.626
Vitamin C (mg)	92.4 ± 2.0	92.0 ± 2.4	96.9 ± 6.4	82.9 ± 9.9	0.519
Vitamin D (mcg)	5.6 ± 0.2 (n = 1116)	5.6 ± 0.2 (n = 905)	5.7 ± 0.6 (n = 161)	4.6 ± 0.5 (n = 50)	0.141
Vitamin E (mg)	7.4 ± 0.1	7.5 ± 0.2	7.3 ± 0.3	6.6 ± 0.4	0.117
Alpha carotene (mcg)	472.5 ± 28.2	486.9 ± 29.6	431.5 ± 66.4	294.7 ± 77.7	0.072
Selenium (mcg)	105.3 ± 1.3	105.6 ± 1.3	103.8 ± 2.8	106.9 ± 7	0.679
Lycopene (mcg)	5447.5 ± 291.4	5452.1 ± 309.1	5538.8 ± 698.8	4964.3 ± 819.1	0.824
Lutein + zeaxanthin (mcg)	1548.9 ± 81.6	1559.6 ± 89.0	1504.2 ± 154.5	1473.2 ± 233.5	0.886
Beta-cryptoxanthin (mcg)	133.5 ± 5.6	132.8 ± 6.7	147.9 ± 23.2	92.9 ± 15.1	0.057
Folate, DFE ^c^ (mcg)	427.7 ± 6.3	429.2 ± 7.3	430.5 ± 24.2	377.8 ± 20.5	0.055

^a^ Weighted mean± standard error based on the NHANES sampling weights. ^b^ Compared antioxidant levels among the three PSA groups using the univariate weighted multinomial logistic model. ^c^ RAE: Retinol activity equivalents, DFE: Dietary folate equivalents.

**Table 5:** Antioxidants associated with PSA status for older men aged ≥65.

	Unadjusted ^a^		Adjusted ^a^	
Factors (Unit)	PSA 4–10 vs. PSA < 4 OR (95% CI)	PSA ≥ 10 vs. PSA < 4 OR (95% CI)	PSA 4–10 vs. PSA < 4 OR (95% CI)	PSA ≥ 10 vs. PSA < 4 OR (95% CI)
**Endogenous antioxidants**				
Bilirubin (umol/L)	1.00 (0.98, 1.02)	0.98 (0.98, 1.02)	1.00 (0.98, 1.03)	0.99 (0.95, 1.02)
Albumin (g/L)	0.99 (0.94, 1.05)	0.88 (0.84, 0.92) ***	1.01 (0.95, 1.07)	0.90 (0.85, 0.96) ***
Uric acid, per 10 umol/L	1.01 (0.99, 1.03)	1.02 (0.97, 1.08)	1.01 (0.99, 1.03)	1.02 (0.97, 1.07)
**Dietary antioxidants**				
Vitamin A, RAE ^b^, per 100 mcg	1.00 (0.98, 1.02)	0.93 (0.87, 1.00) ^#^	1.00 (0.98, 1.02)	0.94 (0.88, 1.01)
Vitamin B2 (mg)	0.98 (0.79, 1.21)	0.85 (0.61, 1.21)	1.02 (0.82, 1.26)	0.96 (0.71, 1.31)
Vitamin C (mg), per 10 mg	1.01 (0.98, 1.04)	0.98 (0.93, 1.03)	1.01 (0.98, 1.04)	0.98 (0.94, 1.03)
Vitamin D (mcg)	1.00 (0.96, 1.05)	0.94 (0.87, 1.02)	1.01 (0.96, 1.05)	0.96 (0.89, 1.03)
Vitamin E (mg)	0.99 (0.95, 1.03)	0.94 (0.88, 1.01)	1.00 (0.96, 1.04)	0.97 (0.90, 1.04)
Alpha-carotene, per 100 mcg	0.99 (0.97, 1.02)	0.95 (0.89, 1.02)	0.99 (0.97, 1.02)	0.96 (0.90, 1.02)
Selenium (mcg), per 10 mcg	0.99 (0.95, 1.02)	1.01 (0.94, 1.08)	1.00 (0.97, 1.03)	1.03 (0.97, 1.10)
Lycopene, per 1000 mcg	1.00 (0.98, 1.02)	0.99 (0.96, 1.02)	1.01 (0.99, 1.03)	1.00 (0.97, 1.03)
Lutein + zeaxanthin, per 1000 mcg	0.99 (0.94, 1.05)	0.99 (0.91, 1.07)	0.99 (0.94, 1.04)	0.98 (0.91, 1.06)
Beta-cryptoxanthin, per 100 mcg	1.03 (0.94, 1.13)	0.83 (0.66, 1.05)	1.03 (0.94, 1.14)	0.83 (0.66, 1.04)
Folate, DFE ^b^, per 100 mcg	1.00 (0.89, 1.13)	0.87 (0.75, 1.00) *	1.03 (0.91, 1.15)	0.91 (0.79, 1.04)

^#^ 0.05 < *p*-value < 0.1; *: *p* < 0.05, ***: *p* < 0.001 based on multinomial logistic models. ^a^ weighted multinomial logistic model with and without adjusting for age; OR: Odds ratio; CI: Confidence interval. ^b^ RAE: Retinol activity equivalents, DFE: Dietary folate equivalents.

## Data Availability

The open-access deidentified NHANES data can be found using the link: https://wwwn.cdc.gov/nchs/nhanes/, accessed on 1 July 2021.

## Supplementary Materials

The following supporting information can be downloaded at: https://www.mdpi.com/article/10.3390/nu15153298/s1, Table S1: Participants’ demographic and behavioral factors associated with PSA status for middle-aged men aged 40–64.9; Table S2: Participants’ demographic and behavioral factors associated with PSA status for older men aged ≥65.
