# Supplementary material for: Dietary and Serum Antioxidants Associated with Prostate-Specific Antigen for Middle-Aged and Older Men
Source: Nutrients. 2023 Jul 25;15(15):3298. doi: 10.3390/nu15153298 (PMC10420876; doi:10.3390/nu15153298)
Supplement: Supplementary file 1 [file nutrients-15-03298-s001.zip › nutrients-2522594-supplementary.pdf]

Table S1. Participants' demographic and behavioral factors associated with PSA status for middle-aged men aged 40-64.9

	Unadjusted[a]		Adjusted[a]	
Factor	PSA 4-10 vs. PSA<4<br>OR (95% CI)	PSA≥10 vs. PSA<4<br>OR (95% CI)	PSA 4-10 vs. PSA<4<br>OR (95% CI)	PSA≥10 vs. PSA<4<br>OR (95% CI)
Age	1.19 (1.13, 1.24)***	1.05 (0.97, 1.14)	1.19 (1.13, 1.25)***	1.07 (0.98, 1.16)
Race				
Non-Hispanic white	1	1	-	-
Non-Hispanic Black	0.92 (0.53, 1.59)	7.09 (1.65, 30.49)**		
Other	1.04 (0.61, 1.77)	3.78 (0.83, 17.14)		
Income to poverty ratio				
≤1	1	1	1	1
1.1- 4	1.11 (0.54, 2.29)	0.08 (0.02, 0.32)***	0.86 (0.41, 1.81)	0.07 (0.02, 0.3)***
>4	1.88 (0.92, 3.84)	0.17 (0.04, 0.72)*	1.43 (0.67, 3.08)	0.15 (0.03, 0.66)*
BMI status				
<25	1	1	-	-
25-29.9	1.22 (0.68, 2.2)	2.41 (0.45, 12.86)		
≥30	0.71 (0.4, 1.24)	1.51 (0.24, 9.44)		
Smoking				
Never	1	1	-	-
Former	1.08 (0.67, 1.76)	0.18 (0.03, 1.22)		
Current	0.61 (0.32, 1.16)	0.82 (0.18, 3.66)		
Days of alcohol usage in the past 12 months			-	-
≤3	1	1		
4-50	0.39 (0.17, 0.91)*	0.48 (0.09, 2.52)		
>50	0.53 (0.27, 1.06)	0.27 (0.05, 1.57)		

*: p<0.05, **p<0.01; ***:p<0.001

[a] weighted multinomial logistic model; OR: odds ratio; CI: confidence interval

Table S2. Participants' demographic and behavioral factors associated with PSA status for older men aged ≥65

	Unadjusted[a]		Adjusted[a]	
Factor	PSA 4-10 vs. PSA<4 OR (95% CI)	PSA≥10 vs. PSA<4 OR (95% CI)	PSA 4-10 vs. PSA<4 OR (95% CI)	PSA≥10 vs. PSA<4 OR (95% CI)
Age	1.04 (1.01, 1.08)**	1.07 (1.01, 1.13)*	1.05 (1.01, 1.08)**	1.07 (1.01, 1.14)*
Race				
Non-Hispanic white	1	1	1	1
Non-Hispanic Black	1.71 (1.19, 2.44)**	2.86 (1.5, 5.44)**	1.82 (1.24, 2.66)**	3.17 (1.6, 6.27)***
Other	0.8 (0.49, 1.32)	1.39 (0.68, 2.83)	0.88 (0.52, 1.48)	1.62 (0.77, 3.39)
Income to poverty ratio				
≤1	1	1	-	-
1.1- 4	0.73 (0.46, 1.14)	1.35 (0.59, 3.09)		
>4	0.61 (0.35, 1.06)	1.35 (0.46, 3.93)		
BMI status				
<25	1	1	-	-
25-29.9	0.75 (0.53, 1.08)	0.58 (0.31, 1.06)		
≥30	0.55 (0.35, 0.86)**	0.62 (0.29, 1.31)		
Smoking				
Never	1	1	-	-
Former	0.74 (0.52, 1.06)	0.61 (0.31, 1.19)		
Current	0.86 (0.47, 1.58)	0.59 (0.23, 1.48)		
Days of alcohol usage in the past 12 months			-	-
≤3	1	1		
4-50	0.92 (0.54, 1.54)	1.02 (0.34, 3.06)		
>50	1.31 (0.79, 2.17)	1.14 (0.44, 2.96)		

*: p<0.05, **p<0.01; ***:p<0.001

[a] weighted multinomial logistic model; OR: odds ratio; CI: confidence interval
